# Disparities in occupational health services: an international comparative study

**DOI:** 10.1186/s12995-023-00386-2

**Published:** 2023-09-14

**Authors:** Ayala Olga Krakov, Oren Zack, Oren Y. Sagiv, Dan Slodownik, Rachel Raanan, Deborah Alperovitch-Najenson, Lilah Rinsky‑Halivni, Shlomo Moshe

**Affiliations:** 1 The Environmental and Occupational Department, School of Public Health, Faculty of Medicine, Tel Aviv University, Tel Aviv, Israel; 2 The Department of Occupational Medicine, Jerusalem and HaShfela District, Maccabi Healthcare Services, Rishon Letzion, Israel; 3 The Department of Dermatology, Tel Aviv Sourasky Medical Center, Faculty of Medicine, Tel Aviv, Israel; 4 The Public Health Services, Ministry of Health, Jerusalem, Israel; 5 The Department of Physical Therapy, Recanati School for Community Health Professions, Faculty of Health Sciences, Ben-Gurion University of the Negev, Beer-Sheva, Israel; 6 The Department of Occupational Medicine, Jerusalem District, Clalit Health Services, Jerusalem, Israel; 7 Department, Braun School of Public Health, Family Medicine, Hadassah-Hebrew University Medical School, Jerusalem, Israel; 8 Harvard T.H. Chan School of Public Health, Boston, USA

**Keywords:** Occupational health services, Occupational physicians, Disparity in occupational medicine

## Abstract

**Background:**

Occupational Health Services (OHS) are comprehensive, multidisciplinary services supplied by various trained workers, including occupational physicians (OP), whose specialty is focused on workers’ health.

**Aims:**

Our study questions are whether the OP/worker ratio may reflect the scope and availability of OHS.

**Methods:**

This comparative study, conducted on globally different OHS, was based on literature analysis of peer-reviewed articles published in journals covering topics of occupational medicine and public health that addressed parameters on the type and scope of OHS provision.

**Results:**

We described the number of OP/worker ratio, and the correlation to economic parameters (Gross domestic product—GDP, health expenditure, Gini Index—GI) by country. We found that among countries with a GDP per capita higher than US$30,000, only three (US, South Korea and Israel) had a very low OP/worker ratio (about 1:50,000 compared to 1:5,000 in other countries). Looking at all the countries together, there was a statistically significant negative correlation between health expenditure percentage of GDP per capita and OP/worker ratio (rs = -0.54, p = 0.01) and a significant positive correlation between GI and OP/worker ratio (rs = 0.47, p = 0.04).

**Conclusions:**

The lesser the percentage of health expenditure of GDP and the subsequent greater general inequality as reflected by the GI, the lower the OP/worker ratio. The data collected in our comparative study show that the OP/worker ratio is a parameter both easy to define and obtain which best represents the status and disparity of OHS in each country.

**Supplementary Information:**

The online version contains supplementary material available at 10.1186/s12995-023-00386-2.

## Introduction

In 1994, the World Health Organization (WHO) adopted a global strategy for "Occupational Health for All", regarding it as a realistic, long-term objective to provide all workers access to competent Occupational Health Services (OHS), irrespective of age, gender, nationality, type of employment, or size and location of the workplace [1]. OHS are comprehensive, multi-disciplinary services supplied by various trained workers, including occupational physicians (OP)—physicians, whose specialty is focused on workers’ health, and have typically finished a residency in occupational medicine. The OP scope of practice includes, among others, disability management and work capacity evaluation (Fitness for Work – FFW), health-hazard evaluation, periodic health surveillance, toxicology counseling, return-to-work, and workers’ compensation case management [2]. The exact nature and the scope of OHS that an organization requires depend on its size, the hazards and risks of the activities in which it is engaged, and legal aspects. Some organizations find it beneficial to purchase or share in OHS [3].

In 2007, Westerholm et al. [3] described OHS in eleven countries. They found that, in most countries, employers are obliged by law to provide OHS for their employees. This obligation is strictly enforced in Finland, France, and Germany and still exists – albeit in a diluted form – in the Netherlands. In specified sectors in Austria, Japan, Norway, and the Czech Republic it is partially applied with various exemptions for companies of a certain size. Despite much of the research that has been published assessing different OHS in many countries [4–20], only two papers supplied cross-country comparisons [3, 21]. This data show the difficulties of publishing such a research on one hand, and on the other hand shows the need for a new study in this field.

The primary question governing all studies is whether there is any suitable method to compare OHS between different countries. Many studies have reported associations between economic variables and different topics. For instance, the relationships between economic conditions and road-traffic accident mortality rates were investigated in 30 member and five accession countries of the Organisation for Economic Cooperation and Development (OECD) [22]. One of the parameters which may reflect the availability of OHS is the OP to workers ratio (OP/workers). National OP/workers ratios were randomly published by various authors from several countries in different years. Our study questions are whether the OP/workers ratio may reflect the scope and availability of OHS, whether there are prominent differences among countries, and whether the reason for differences among OHS lies in social inequality, regulator prioritization, or economic priorities.

## Methods

This international comparative study, conducted on globally different OHS, was based on a literature review of peer-reviewed articles published in journals covering topics of occupational medicine and public health that addressed parameters on the type and scope of OHS provision. We initiated a general search strategy using PubMed and Google Scholar for English papers, published between 2000 to 2020, that provided data on OHS in different countries. The literature search using the term “Number of occupational physicians” gave 2,485 results in PubMed and 1,040,000 results in Google Scholar. We further used the following search terms: “occupational medicine status”, “occupational inequality”, “occupational health inequity”, “worker health inequality”, “number of OP in each country”, “occupational medicine disparities”, and “occupational medicine services”. All together we found about 150 relevant publications. Lastly, we used these search terms by any of the specific countries that matched the initial search with relevant articles. The inclusion criteria were review or original articles that thoroughly detailed the status of OHS in the respective countries, for example, included services (preemployment health examination, periodical occupational health examination, and FFW evaluation), availability of OHS, number of OP employed, etc. Eighty-seven articles met the above criteria. Articles that did not report basic data like the number of OP, the scope of services, availability of OHS, etc. were excluded. We preferred the most updated publication in each country. All together we choose 20 countries with enough data, as needed by the mentioned criteria (Chart 1).

To find whether the scope of OHS truly represents disparities, we decided to compare the scope of occupational medicine services and the OP/worker ratios to sociodemographic parameters. The dependent parameter is the OP/workers ratio in each country. This figure represents how many workers are serviced by one OP. We assumed that the higher the ratio (1:3,000 is higher than 1:5,000), the better and more available are the OHS. The independent variables included:1. The Gross domestic product (GDP) per capita, which is the standard measure of the value added by producing goods and services in a country during a specific period divided by the number of citizens [23]. As such, GDP also measures the income earned from that production or the total amount spent on final goods and services; 2. Health expenditure per person—the result of GDP per capita multiplied by the percentage of GDP dedicated to health; 3. The Gini index (GI)—this coefficient is based on the comparison of cumulative proportions of the population against cumulative proportions of income they receive, and it ranges between 0 in the case of perfect equality and 1 in the case of perfect inequality [24]. The World Bank Group database were matched to publication year of each study. All data was obtained from the OECD web site [23] and World bank group [24].

The scope of OHS was evaluated using three types of medical services which are common in OHS:

1. Periodic occupational medical surveillance of workers exposed to specific hazards – this procedure is carried out based on environmental monitoring and occupational health regulations, at regularly scheduled intervals, and includes specific medical screening tests when warranted; 2. Pre-employment health assessment – meaning the referral of a new employee to an occupational medicine clinic before starting work; 3. Fitness for work assessment—defined as determining whether a patient is fit to perform his or her duties without risk to oneself and/or others 4. General Practitioner services – meaning that the OP gives primary medical care to the workers [17].

We used SPSS Version: 1.0.0.1406 in the statistical analysis. Prior to performing data analysis, Kolmogorov–Smirnov tests of normal distribution were computed. Since the distribution was not normal, Spearman and Mann–Whitney U tests were carried out to test for correlation and differences between variables. In addition, multiple logistic regression analysis was performed on the same group of countries to model the relationship between the OP/workers ratio, the GI, and economic variables. A p-value of less than or equal to 0.05 was considered statistically significant.

The study was approved by the Maccabi Health Services institutional review board. The ethics committee waived the need for formal approval since data was collected from publications (Reviews and original research).

## Results

Table 1 describes the number of workers and OP, the OP/workers ratio, and the economic parameters (GDP, health expenditure, GI) by country. There are noticeable differences between countries. For instance, the GDP per capita in Nigeria was US$1,891 with 90 OP, while in Norway, the GDP per capita was US$85,139 with 2,523 OP. We found that among countries with a GDP per capita higher than US$30,000, only three (US, South Korea and Israel) had a very low OP/worker ratio (about 1:50,000 compared to 1:5,000 in other countries).

**Table 1:** No of Workers, Occupational Physicians, GDP, Health expenditure and GINI index by country

Country	No of Workers (Thousands)	OP	Ratio OP/worker	GDP **per capita**	Health expenditure	GINI Index
Nigeria	51,778	90	575,311	1,891	68	0.43
Brazil	95,333	10,000	9,533	8,642	726	0.54
Mexico	55,131	255	216,200	9,287	512	0.45
CR*	5,185	500	10,370	18,466	1,110	0.26
Spain	23,034	5,500	4,188	29,600	2,655	0.35
South Korea	28,809	540	53,350	42,998	3,509	0.31
Italy	25,986	9,856	2,637	32,407	2,810	0.36
Japan	65,639	83,000	791	48,603	5,250	0.33
Germany	41,542	12,787	3,249	38,432	3,939	0.28
Austria	4,202	2,000	2,101	39,384	3,753	0.28
Australia	11,254	270	41,681	40,095	3,308	0.33
France	26,376	5,694	4,632	40,144	4,649	0.29
Finland	2,540	2,369	1,072	40,873	3,916	0.26
UK	34,129	1,207	28,276	46,372	4,549	0.35
Israel	3,967	90	44,078	42,912	3,218	0.35
Netherland	8,290	2,400	3,454	45,079	4,575	0.29
Sweden	4,907	1,262	3,888	46,946	4,117	0.27
Denmark	2,931	191	15,345	53,254	5,443	0.28
USA	164,268	4,725	34,766	57,951	9,875	0.41
Norway	2,523	340	7,421	85,139	6,854	0.27

^*^CR Czech Republic, *UK* United Kingdom, No of Workers—Workers, Labor force by thousands, *OP* Occupational Physicians, Ratio of Workers per OP, GDP—Gross domestic product‏ – per capita, US$; Health expenditure—Health expenditure of GDP per capita, US$

Table 2 describes the scope of OHS by country and by type of service. Once again, a considerable cross-country variation exists: occupational health surveillance is considered a basic service and that is why it is performed in all countries, while FFW evaluations and Pre-employment examination are done according to legislation and consequently carried out in only about half of the countries surveyed.

**Table 2:** Types of Occupational Health Services (Pre-Employment examination, Occupational periodic health examination, Fitness For work examination, and General Practitioner services) by country

Country	Pre-Employment examination	Occupational periodic health examination	Fitness For work examination	General Practitioner services
Nigeria		✔		✔
Brazil		✔		
Mexico	✔	✔		
CR	✔	✔	✔	
Spain	✔	✔	✔	✔
South Korea	✔	✔	✔	✔
Italy	✔	✔	✔	✔
Japan		✔		✔
Germany	✔	✔	✔	✔
Austria		✔		✔
Australia		✔	✔	✔
France	✔	✔	✔	
Finland	✔	✔	✔	✔
UK	✔	✔	✔	
Israel	✔	✔	✔	
Netherland		✔		✔
Sweden		✔		✔
Denmark	✔	✔		✔
USA		✔		✔
Norway		✔		

We expected low GDP to indicate poorer access to OHS and decided to divide countries by GDP per capita to < US$20,000 (low) and > US$20,000 (high). An annual GDP per capita of US$20,000 is considered a good point of departure between developing and developed countries [23]. Using a Mann–Whitney U test we determined if the differences in the OP/workers ratio between high and low GDP countries affected the provision of FFW services. The median for the two groups was not statistically significant (U = 50, z = 0, *p* = 1) results not shown.

Table 3 presents the linear regression between OP/workers ratio and several macro-economic variables in all countries and countries with GDP per capita > US$20,000. In countries with GDP per capita > US$20,000, there was no significant correlation between their OP/workers ratio and GDP per capita, health expenditure percentage of GDP, health expenditure per capita, and GI. However, looking at all countries together, there was a significantly negative correlation between health expenditure of GDP per capita and OP/workers ratio (rs = -0.33, *p* = 0.02), health expenditure percentage of GDP per capita and OP/workers ratio (rs = -0.54, *p* = 0.01) and a significant positive correlation between GI and OP/worker ratio (rs = 0.47, *p* = 0.04).

**Table 3:** The linear regression between Ratio of Workers per OP and GDP per capita, Health expenditure and GINI index by country

	All Countries		Countries with GDP **per capita** > **US$**20.000	
Parameters	Correlation Coefficient	p	Correlation Coefficient	p
GDP	-0.15	0.53	0.26	0.34
Health expenditure percent of GDP	-0.54	*0.01	-0.34	0.19
Health expenditure	-0.33	*0.02	-0.02	0.96
GINI index	0.47	*0.04	0.36	0.17

^*^p < 0.05

The results in Table 4 were obtained from a multiple linear regression analysis of the OP/worker ratio with three economic variables: GDP per capita, GI, and health expenditure percentage in the same group of countries. When run on countries with GDP per capita > US$20,000, the variation of the model was statistically significant (*p* = 0.04) but none of the independent variables contributed significantly to the total variance. When all countries were included in the analysis the tendency of all parameters together became significant (*F* = 4.4, *p* = 0.02).

**Table 4:** Results of Multiple Linear Regression Analysis of various variables with OP/Workers ratio

**Countries with GDP per capita > US$20,000**					
	Regression Coefficient (β)	Standard Error	t	p	Standardized Coefficient
GDP	0.30	0.36	0.84	0.42	0.21
Health expenditure percent of GDP	-2,662	2,220	-1.20	0.25	-0.32
GINI index	273,123	115,907	2.36	0.04	0.64
*F* = 1.88, *p* = 0.19					
**All Countries**					
GDP	-0.82	1.98	-0.41	0.69	-0.12
Health expenditure percent of GDP	-22,417	11,485	-1.95	0.07	-0.45
GINI index	546,750	418,176	1.31	0.21	0.31

*F* = 4.4, *p* = 0.02

## Discussion

Our study aimed to clarify the reasons behind differences in OHS among these countries. In order to achieve this goal we compared the OP/workers ratio and the scope of OHS provision in 20 countries with their corresponding macro-economic indicators of GDP per capita, health expenditure per capita, and GI. We found a significantly negative correlation between health expenditure percentage of GDP and OP/workers ratio. Additonally, significant positive correlation was found between the GI and the OP/workers ratio. Negative correlation means the higher health expenditure percentage less workers are treated with one OP, meaning higher rate of OP/workers. Positive correlation means that higher GI (inequality) correlates with more workers per one OP, meaning lower rate of OP/workers.

There are some drawbacks to our study. We got enough data for only 20 countries. Usually reports come from developed countries so it might give bias status considering the status of OHS. There are difference among countries like the industry mix of the country’s economy or OHS regulatory/legal system which could influence the OP/workers rate. Other professions within OHS like nurses, ergonomists, hygienists, employee support, health and safety managers etc. were not considered. The number of OP is relevant to the year of publication.

To the best of our knowledge, no other publication used these parameters to evaluate OHS between countries. Radon et al. [21] compared different parameters of OHS among eighteen countries including the fatality rate of work-related accidents, accident insurance systems, and workers' compensation in case of an occupational accident or the coverage of occupational disease by the workers' compensation insurance. We think that these parameters are easy to trace but do not represent the full scope of OHS, but rather, the government’s actions concerning workers’ safety, enforcement of safety regulations, etc. We believe the workers' compensation system represents the policy of national insurance and private companies in each country and not necessarily the scope of OHS.

Westerholm et al. [3] compared eleven OHS. They found that the countries which strictly enforce by law the provision of OHS can, for obvious reasons, demonstrate high rates of workforce coverage, approaching 100%. We agree this is a good parameter (representing the availability of OHS but not necessarily the quality). However, in most countries, it is difficult to conduct reliable assessments due to statistical uncertainties—in the UK, Japan, Austria, Sweden, and the Czech Republic estimates were particularly imprecise [3]. In most countries in this review, the funding of OHS was supported by client companies and employers. Radon et al. [21] showed that in the majority of the 18 countries represented in their research, the premiums for OHS were paid only by the employer and hinged on the risk and size of the enterprise. After reviewing the literature, we conclude that, in almost all countries, OHS are paid by the employer, thus making the parameter of OHS funding immaterial in trying to com-pare services between different countries.

The National Institute for Occupational Safety and Health (NIOSH) established some strategic goals that best represent the health and safety issues facing the US workforce [25]. Among these goals are reducing the rates of occupational cancer, cardiovascular disease, occupational hearing loss, occupational musculoskeletal disorders, etc. There are several difficulties in converting these strategic goals to parameters that could evaluate OHS. An occupational disease registry could help monitor and evaluate trends in occupational disease as outlined above. However, in most countries, these registries are voluntary and do not represent true rates. Another difficulty would be the latency period for an occupational disease which could take 10–20 years to manifest. Other goals depend on the employer's attitude towards occupational safety and health. In light of the above, we believe these parameters are difficult to trace and analyze in most countries, due to their subjectivity and wide variability and would not be used as a good monitor for OHS.

We also believe that all parameters presented thus far are prone to be influence by many factors such as employer attitude, safety regulations, degree of enforcement, insurance legislation, etc. When we compared different services of OHS we found that FFW differentiate better since pre-employment examination service and periodic health examination service is given in all countries, and only FFW services is given in several countries, definitely not in many, as other service. However, whether FFW evaluation is given does not explain well the gap among different OHS.

Consequently, we propose the OP/workers ratio as an easily measured and simplified method of comparing OHS among different countries. This notion is not entirely novel. In 2002, Rantanen et al. [26] published a review on OHS in Europe. They found that the ratio of OP/workers and occupational health nurses/workers in Europe varies between 1 per 500 and 1 per 5,000. Nicholson [27] considered the challenges facing occupational medicine in the UK and how to improve access to OHS in 2004, and concluded that there are no readily obtainable comparable data particularly concerning full-time/part-time status, practicing/retired members, and level of qualification/training thus making this benchmarking difficult. We postulate that currently available data in the medical literature and other online resources make it easier to benchmark.

The primary debate is how much of the data found in our study truly represent the status of OHS. We were not able to evaluate the quality of OHS since quality is difficult to measure and data is missing from most country registries. We found that the scope of basic OHS is different between countries, but that this difference is not explained by the disparity in OP/workers ratio. Nonetheless, we propose to focus on the status of three countries with high GDP/capita and low workers/OP ratio (South Korea, Israel, and the USA) and examine whether a low ratio truly represents disparities in OHS in these countries.

The rate of OP/workers in South Korea was 1:53,350. South Korea OHS are provided mainly by private OHS institutions outside the workplace. Very few workplaces have their own services. Occupational medical examinations for workers exposed to potential work hazards must be provided within paid working hours at the employers expense [10]. However, most employers in small businesses tend to ignore or neglect the OHS because they have limited resources than large businesses. In 2011, the South Korean government launched regional public Workers Health Centers to provide small businesses with basic OHS. These centers are staffed by OP and are free of charge. In 2019 approximately 20 workers health centers are operating. According to the South Korean labor force there is an urgent need to extend this program [11].

The rate of OP/workers in Israel was 1:44,078. In Israel, in contrast to other developed countries, OHS are socialized and available to every employee in the country under the National Health Insurance [17]. The OHS are anchored by legislation; some services such as health surveillance of workers exposed to specific hazards are required of employers to be performed by law, while other services are essentially a given right to every worker. Research in the field of occupational medicine in Israel is currently scant [17]. Contrary to the norm in most Western countries, there is currently no National Institute of Occupational Health in Israel that focuses on environmental and occupational health in the workplace. Another concern is outdated occupational standards and regulations that were last updated in the 1990s. Consequently, many sectors like the agriculture and construction industries, and small workplaces with less than 50 workers, are overlooked.

Baker et al. [19] reported, based on demographic data provided by the American College of Occupational and Environmental Medicine (ACOEM), that in 2007, 4,725 physicians were registered ACOEM members leading to a respective OP/workers ratio of 1:34,766 (Table 1). We did not find the coverage rate of the US working population, but we assume it is probably the same than the UK (12%). Also, the OHS in US includes only pre-employment and periodic health examination. The low availability may manifest in high occupational injury and illness rates. Injuries at work comprise a substantial part of the country's injury burden, accounting for nearly half of all injuries in some age groups [28]. Souza et al. [29] evaluated different parameters to estimate disparities in occupational health in the US. They suggested parameters like work fatalities, work accidents, work-related disease, health claims data, etc. We have explained why many traditional parameters such as these are less accurate and multi-factorial, and hence better not be used to estimate occupational health.

Surveillance of a health disparity necessarily involves defining the disparity and deciding how progress towards reducing it will be measured. We think the data collected in our research show that the OP/workers ratio is a parameter both easy to define and can be obtain which best represents the availibility of OHS in either country, as it correlates with GDP per capita, Health expenditures and GI. The implications of the data collected in this study are that we are still far from fulfillment of the concept of “Occupational Health for All”, and that there is much to be done in this field. Last but not least is the question of what should be the ultimate ratio. In developed countries (GDP per capita > US$30,000) it seems that an OP/workers of 1:5000 is a reasonable and acceptable ratio to support proper and accessible OHS to the working population.

### Supplementary Information

**Additional file 1.** Search results.

## Data Availability

The data is kept in the MHS database and will be available under demand.

## Abbreviations

WHO: World Health Organization
OHS: Occupational Health Service
OP: Occupational Physician
GI: Gini index
FFW: Fitness for Work
NIOSH: National Institute for Occupational Safety and Health
ACOEM: American College of Occupational and Environmental Medicine
