# Supplementary material for: Disparities in occupational health services: an international comparative study
Source: J Occup Med Toxicol. 2023 Sep 14;18:21. doi: 10.1186/s12995-023-00386-2 (PMC10503138; doi:10.1186/s12995-023-00386-2)
Supplement: Supplementary file 1 — Additional file 1. Search results. [file 12995_2023_386_MOESM1_ESM.docx]

**Flow Chart 1:** Search results

| Published articles with key word:  "Number of occupational physicians"  **(n=2485)** |
| --- |

| Published articles with key word:  Number of occupational physicians + occupational medicine status  **(n=139)** |
| --- |

| Published articles with key word:  Number of occupational physicians + occupational medicine services +  **(n=382)** |
| --- |

| Published articles with key word: worker health inequality + occupational medicine status(n=151) |
| --- |

| Published articles with key word: worker health inequality + occupational medicine status + some of the inclusion criteria - (n=78) |
| --- |

| Published articles with key word: worker health inequality + occupational medicine status + inclusion criteria - detailed the status of OHS in the respective countries(n=21) |
| --- |
